# Anticipated time to seek medical advice for possible ovarian cancer symptoms and perceived barriers to early presentation among Palestinian women: a national cross-sectional study

**DOI:** 10.1186/s12885-023-11484-z

**Published:** 2023-10-13

**Authors:** Mohamedraed Elshami, Sondos Al-Madhoun, Mohammed Alser, Ibrahim Al-Slaibi, Areej Yaseen, Aya Tuffaha, Hadeel Jabr, Sara Ubaiat, Salma Khader, Reem Khraishi, Inas Jaber, Zeina Abu Arafeh, Aya Alqattaa, Asmaa Abd El Hadi, Ola Barhoush, Maysun Hijazy, Tamara Eleyan, Amany Alser, Amal Abu Hziema, Amany Shatat, Falasteen Almakhtoob, Balqees Mohamad, Walaa Farhat, Yasmeen Abuamra, Hanaa Mousa, Reem Adawi, Alaa Musallam, Shurouq I. Albarqi, Nasser Abu-El-Noor, Bettina Bottcher

**Affiliations:** 1 Division of Surgical Oncology, Department of Surgery, University Hospitals Cleveland Medical Center, 11100 Euclid Avenue, Lakeside 7100, Cleveland, OH 44106 USA; 2 Ministry of Health, Gaza, Palestine; 3 Al-Shifa Hospital, Gaza, Palestine; 4 United Nations Relief and Works Agency for Palestine Refugees (UNRWA), Gaza, Palestine; 5 Almakassed Hospital, Jerusalem, Palestine; 6 Faculty of Medicine, Al-Quds University, Jerusalem, Palestine; 7 Al-Watani Hospital, Nablus, Palestine; 8 Faculty of Medicine, Al-Quds University, Bethlehem, Palestine; 9 Faculty of Medicine, An-Najah National University, Nablus, Palestine; 10 Faculty of Medicine, Islamic University of Gaza, Gaza, Palestine; 11 Faculty of Medicine, Palestine Polytechnic University, Hebron, Palestine; 12 Doctors Without Borders (Medecins Sans Frontieres), Hebron, Palestine; 13 Faculty of Medicine, Al-Quds University, Jenin, Palestine; 14 Faculty of Medicine, Al-Azhar University of Gaza, Gaza, Palestine; 15 Al-Aqsa Hospital, Deir Albalah, Palestine; 16 Faculty of Pharmacy, Al-Azhar University of Gaza, Gaza, Palestine; 17 Faculty of Nursing, Islamic University of Gaza, Gaza, Palestine

**Keywords:** Ovarian cancer, Symptom awareness, Early presentation, Barriers, Seeking medical advice, Palestine

## Abstract

**Background:**

Several factors contribute to delayed presentation with ovarian cancer (OC) symptoms including poor symptom awareness and barriers to seeking help. This study explored the anticipated time to seek medical advice for possible OC symptoms and its association with OC symptom awareness. In addition, it examined perceived barriers that may delay help-seeking among Palestinian women.

**Methods:**

A cross-sectional study was conducted among adult women (≥ 18 years) recruited from hospitals, primary healthcare centers, and public spaces in 11 Palestinian governorates. A modified version of the OC awareness measure was used to collect data in face-to-face interviews. The questionnaire comprised three sections: sociodemographic details, awareness of 11 OC symptoms and time to seek medical advice, and barriers to early presentation.

**Results:**

Of 6095 participants approached, 5618 completed the OCAM (response rate = 92.1%). The proportion of participants who would immediately seek medical advice for a possible OC symptom varied based on the symptom’s nature. For OC symptoms with pain, the proportion that reported immediate seeking of medical advice ranged from 7.9% for ‘persistent low back pain’ to 13.6% for ‘persistent pain in the pelvis’. For non-specific potential OC symptoms, the proportion that reported immediate seeking of medical advice ranged from 2.3% for ‘feeling full persistently’ to 15.8% for ‘increased abdominal size on most days’. Good OC symptom awareness was associated with higher likelihood of seeking medical advice within a week from recognizing 10 out of 11 OC symptoms.

Emotional barriers were the most common barriers with ‘feeling scared’ as the most reported barrier (*n* = 1512, 52.4%). Displaying good OC symptom awareness was associated with a lower likelihood of reporting ≥ 4 emotional barriers (OR = 0.61, 95% CI: 0.38–0.98).

**Conclusion:**

Participants with good OC symptom awareness were more likely to seek medical advice earlier and to display fewer emotional barriers. Establishing educational interventions to raise OC awareness may help in promoting earlier help-seeking and, thus, facilitate earlier diagnosis and improved prognosis.

**Supplementary Information:**

The online version contains supplementary material available at 10.1186/s12885-023-11484-z.

## Introduction

With 313,959 new cases estimated in 2020, ovarian cancer (OC) is considered the third most common gynecological malignancy worldwide [1]. OC is also the second leading cause of cancer-related mortality among gynecological malignancies with estimated 207,252 deaths in 2020 [1]. In Palestine, OC is the second most common among gynecologic cancers, after corpus uterine cancer, accounting for 1.5% of all cancer cases. About 72.0% of Palestinian women diagnosed with OC eventually die from this disease [2].

Women diagnosed with OC in earlier stages have a better prognosis [3]. Timely medical consultation following the onset of possible OC symptoms is essential for prompt diagnosis and initiation of appropriate treatment [4]. Prior studies demonstrated that diagnosis at late stages could be partly attributed to late presentation, due to low OC symptom awareness [5]. A previous national study from Palestine has demonstrated that only 17.4% of the surveyed women displayed good OC symptom awareness [6]. However, the association between OC symptom awareness and time to seek medical advice has not been examined.

There are several other contributing barriers to delayed symptomatic presentation including old age, low socio-economic status, misinterpreting the seriousness of symptoms, and fears about what might be found [5]. Exploring and addressing such barriers is especially important in countries with low resources like Palestine. By assessing these factors, we can identify gaps in knowledge and awareness, as well as potential barriers that hinder timely medical consultation. Such insights can inform the development of educational campaigns, interventions, and policies aimed at promoting early detection and reducing the burden of OC.

Therefore, this study aimed to (i) describe the anticipated time interval to seek medical advice for a possible OC symptom, (ii) examine the association between OC symptom awareness and the anticipated time to seek medical advice, and (iii) identify the barriers to early presentation and their association with OC symptom awareness.

## Methods

### Study design and population

This cross-sectional study is part of the national project Cancer Awareness in Palestine, which was conducted from July 2019 to March 2020 in 11 governorates across the two primary geographical regions of Palestine: seven in the West Bank and Jerusalem (WBJ) and four in the Gaza Strip [6–16]. The project aimed to assess the public awareness of different types of cancers including breast, colorectal, lung, cervical, and ovarian cancers in the Palestinian community utilizing the appropriate versions of the validated Cancer Awareness Measure [17–21].

For this study, inclusion criteria required participants to be adult women (≥ 18 years), possess Palestinian nationality, and be present at one of the designated study sites. Exclusion criteria were working or studying in a health-related field, holding a nationality other than Palestinian, being a visitor to oncology departments during the data collection period, and inability to complete the questionnaire.

### Sample size calculation

There were 1,534,371 females in Palestine who aged 15 years or older in 2019 [22]. We hypothesized that among study participants, 30% at most would seek immediate medical advice for a possible OC symptom. With a confidence level of 95.0%, a type I error rate of 5.0% and an absolute error of 1.0%, the minimum required sample size to detect a 30% proportion of immediate help seeking for a possible OC symptom was 2098 participants.

### Sampling methods

Convenience sampling was used to recruit participants from governmental hospitals, primary healthcare centers and public spaces, such as malls, parks, mosques, churches, public transport hubs, and others. The inclusion of different geographic locations was meant to maximize the representativeness of the study cohort of the Palestinian community [6–16].

### Questionnaire and data collection

A modified version of the ovarian cancer awareness measure (OCAM) was used as an assessment tool [20]. Two bilingual experts in clinical research and survey design translated the OCAM from English into Arabic. Subsequently, the Arabic version was translated back into English by two other bilingual experts. The translated version was then reviewed by five independent experts in the fields of gynecology, oncology, and public health to assess its content validity and translation accuracy.

For the purposes of this study, the original OCAM was modified. The study questionnaire consisted of three sections. The first section described socio-demographic details. The second section evaluated the participants' awareness of 11 OC symptoms using a 5-point Likert scale (1 = strongly disagree, 5 = strongly agree). The original OCAM included 10 symptoms with yes/no/unknown responses, which were modified into 5-point Likert scale to minimize the possibility of answering questions at random. In addition, ‘unexplained weight loss’ had been used in other cancer awareness measurement tools [18, 19] and it was deemed relevant for inclusion in the context of OC. OC symptom awareness was evaluated using a scoring system as previously described [6–16, 23]. Answers with ‘agree’ or ‘strongly agree’ were considered correct; all other answers were considered incorrect. Each correctly identified OC symptom was given one point. The total awareness score was then calculated, ranging from 0 to 11. Based on the number of symptoms recognized, OC symptom awareness was categorized as poor (0 to 4), fair (5 to 8), or good (9 to 11). The second section also included open-ended questions to assess the anticipated time participants would seek medical advice if they recognized each of the OC symptoms. The third section evaluated perceived barriers to early presentation using the same 5-point Likert scale mentioned earlier.

To assess the clarity of the questions in the Arabic version of the OCAM, a pilot study was conducted with 128 respondents who met the inclusion criteria. Participants from the pilot study were excluded from the final analysis. The internal consistency of the modified OCAM was evaluated using Cronbach's Alpha, which yielded an acceptable value of α = 0.78.

Data collection for this study was conducted using the 'Kobo Toolbox,' an electronic and secure tool that allows for online and offline data collection on mobile devices [24]. Participants were invited to complete the questionnaire through face-to-face interviews with trained data collectors. Specifically, female data collectors were selected and trained on using the electronic data collection tool, approaching potential participants, as well as providing a clear explanation of the study and questionnaire. The choice of female data collectors was intended to facilitate effective communication and create a comfortable environment for study participants when discussing this sensitive subject matter.

### Statistical analysis

Participant characteristics were summarized using descriptive statistics. Continuous variables were described using the mean and standard deviation (SD). Categorical variables were described as frequencies and percentages. In consistence with the original OCAM [20], the age of 45 years was utilized to dichotomize the continuous variable of age in order to reflect the age-related risk of OC: 18–44 years and ≥ 45 years. In addition, the continuous variable of monthly income was dichotomized based on the cutoff of 1450 NIS (about $450) as it is the minimum wage in Palestine [25]. Baseline characteristics of participants from the Gaza Strip and the WBJ were compared using appropriate statistical tests. The two-sample t-test was used for continuous variables, and Pearson's Chi-square test was used for categorical variables.

OC symptoms were categorized into two categories: (i) symptoms with pain and (ii) symptoms of a non-specific nature. The anticipated time needed to seek medical advice for these symptoms was categorized into four main categories: immediately (within 24 h), 2–7 days, > 7 days and never. The time category reported by participants from the WBJ versus those from the Gaza Strip was summarized using frequencies and percentages and compared using Pearson’s Chi-Square test. Multivariable logistic regression analysis was performed to examine the association between OC symptom awareness and the anticipated time to seek medical advice for a possible OC symptom. The multivariable analysis adjusted for age group, educational level, employment status, monthly income, marital status, place of residency, having a chronic disease, knowing someone with cancer, and site of data collection. The factors included in the multivariable analysis were determined a priori based on clinical relevance shown in previous studies [5, 6, 15, 26–31].

Consistent with the original OCAM [20], perceived barriers to early presentation were classified into three categories: emotional, service-related, and practical barriers. The frequency and percentage of participants displaying each barrier category (i.e., responding with 'agree' or 'strongly agree') were reported. A comparison between participants from the Gaza Strip and the WBJ was performed using Pearson's Chi-Square test. The median number of barriers in each category (overall, emotional, service, and practical) was utilized as a cutoff to dichotomize the number of displayed barriers. Multivariable logistic regression analysis was then performed to examine the association between OC symptom awareness and displaying at least the median number of barriers in each category. The same aforementioned multivariable model was used in all those analyses.

A complete case analysis was used to handle missing data, which were hypothesized to be missed completely at random. Stata software, version 17.0 (Stata Corp, College.

Station, Texas, United States) was used in all statistical analyses.

## Results

### Participant characteristics

Out of 6095 approached, a total of 5618 participants agreed to take part in the study and completed the questionnaire (response rate = 92.1%). The final analysis included 5411 questionnaires (3133 from the WBJ and 2278 from the Gaza Strip) since 158 had missing data and 49 were ineligible. Participants from the WBJ were older, had more frequent comorbidities, and gained higher monthly income than participants from the Gaza Strip (Supplementary table 1). However, the likelihood of participants from the Gaza Strip having good awareness of OC symptoms was higher than those from the WBJ (21.0% vs. 14.8%).

### Immediate seeking of medical advice for a possible OC symptom

The proportion of study participants who reported that they would immediately seek medical advice for a possible OC symptom with pain was 7.9% (*n* = 427) for ‘persistent low back pain’, 11.7% (*n* = 633) for ‘persistent pain in the abdomen’, and 13.6% (*n* = 733) for ‘persistent pain in the pelvis’ (Table 1). Participants from the Gaza Strip were more likely than participants from the WBJ to immediately visit a doctor when asked about all OC symptoms with pain except for ‘persistent low back pain,’ where no notable difference was observed.

**Table 1:** Summary of the reported time to seek medical advice for a possible ovarian cancer symptom

Category of symptoms	OC sign/symptom	Total (*n* = 5411) n (%)	Gaza Strip (*n* = 2278) n (%)	WBJ (*n* = 3133) n (%)	*p*-value
**Symptoms with pain**	**Persistent low back pain**				
	Immediately	427 (7.9)	169 (7.4)	258 (8.2)	0.21
	≤ 7 days	3001 (55.5)	1245 (54.7)	1756 (56.0)	
	> 7 days	342 (6.3)	158 (6.9)	184 (5.9)	
	Never	1641 (30.3)	706 (31.0)	935 (29.8)	
	**Persistent pain in the pelvis**				
	Immediately	733 (13.6)	356 (15.6)	377 (12.0)	< 0.001
	≤ 7 days	3781 (69.9)	1573 (69.1)	2208 (70.5)	
	> 7 days	292 (5.4)	121 (5.3)	171 (5.5)	
	Never	605 (11.2)	228 (10.0)	377 (12.0)	
	**Persistent pain in the abdomen**				
	Immediately	633 (11.7)	299 (13.1)	334 (10.7)	< 0.001
	≤ 7 days	3790 (70.0)	1590 (69.8)	2200 (70.2)	
	> 7 days	291 (5.4)	133 (5.8)	158 (5.0)	
	Never	697 (12.9)	256 (11.2)	441 (14.1)	
**Symptoms of a nonspecific nature**	**Extreme generalized fatigue**				
	Immediately	267 (4.9)	121 (5.3)	146 (4.7)	< 0.001
	≤ 7 days	1913 (35.4)	952 (41.8)	961 (30.7)	
	> 7 days	352 (6.5)	117 (5.1)	235 (7.5)	
	Never	2879 (53.2)	1088 (47.8)	1791 (57.2)	
	**Unexplained weight loss**				
	Immediately	479 (8.9)	157 (6.9)	322 (10.3)	< 0.001
	≤ 7 days	1686 (31.2)	660 (29.0)	1026 (32.7)	
	> 7 days	765 (14.1)	388 (17.0)	377 (12.0)	
	Never	2481 (45.9)	1073 (47.1)	1408 (44.9)	
	**Increased abdominal size on most days**				
	Immediately	852 (15.8)	376 (16.5)	476 (15.2)	< 0.001
	≤ 7 days	2950 (54.5)	1334 (58.6)	1616 (51.6)	
	> 7 days	489 (9.0)	183 (8.0)	306 (9.8)	
	Never	1120 (20.7)	385 (16.9)	735 (23.5)	
	**Passing more urine than usual**				
	Immediately	408 (7.5)	191 (8.4)	217 (6.9)	< 0.001
	≤ 7 days	2688 (49.7)	1268 (55.7)	1420 (45.3)	
	> 7 days	299 (5.5)	110 (4.8)	189 (6.0)	
	Never	2016 (37.3)	709 (31.1)	1307 (41.7)	
	**Changes in bowel habit**				
	Immediately	396 (7.3)	174 (7.6)	222 (7.1)	< 0.001
	≤ 7 days	2199 (40.6)	993 (43.6)	1206 (38.5)	
	> 7 days	155 (2.9)	46 (2.0)	109 (3.5)	
	Never	2661 (49.2)	1065 (46.8)	1596 (50.9)	
	**Persistent bloating**				
	Immediately	347 (6.4)	202 (8.9)	145 (4.6)	< 0.001
	≤ 7 days	2092 (38.7)	1037 (45.5)	1055 (33.7)	
	> 7 days	189 (3.5)	81 (3.6)	108 (3.4)	
	Never	2783(51.4)	958 (42.1)	1825 (58.3)	
	**Difficulty eating on most days**				
	Immediately	339 (6.3)	155 (6.8)	184 (5.9)	< 0.001
	≤ 7 days	2146 (39.7)	1019 (44.7)	1127 (36.0)	
	> 7 days	290 (5.4)	104 (4.6)	186 (5.9)	
	Never	2636 (48.7)	1000 (43.9)	1636 (52.2)	
	**Feeling full persistently**				
	Immediately	126 (2.3)	71 (3.1)	55 (1.8)	< 0.001
	≤ 7 days	835 (15.4)	458 (20.1)	377 (12.0)	
	> 7 days	138 (2.6)	70 (3.1)	68 (2.2)	
	Never	4312 (79.7)	1679 (73.7)	2633 (84.0)	

*n* number of participants, *WBJ* West Bank and Jerusalem,* OC* ovarian cancer

The proportion of study participants who reported that they would immediately seek medical advice for a possible OC symptom of a non-specific nature ranged from 2.3% (*n* = 126) for ‘feeling full persistently’ to 15.8% (*n* = 852) for ‘increased abdominal size on most days.’ Participants from the Gaza Strip were more likely than participants from the WBJ to immediately visit a doctor when asked about all non-specific OC symptoms except for ‘unexplained weight loss,’ where the opposite was found.

### Association between OC symptom awareness and no seeking of medical advice

Generally, participants with better OC symptom awareness were more likely to seek medical advice early for all OC symptoms except ‘persistent bloating’ and ‘feeling full persistently’, where no associated differences were found (Table 2).

**Table 2:** Multivariable logistic regression analyzing the association between no seeking of medical advice and awareness of ovarian cancer symptoms

**Awareness of OC symptoms**	**A) Symptoms with pain**							
	**Persistent low back pain**		**Persistent pain in the pelvis**		**Persistent pain in the abdomen**			
	**AOR (95% CI)**	***p*-value**	**AOR (95% CI)**	***p*-value**	**AOR (95% CI)**	***p*-value**		
**Poor**	Ref	Ref	Ref	Ref	Ref	Ref		
**Fair**	0.89 (0.78–1.02)	0.09	0.94 (0.78–1.13)	0.50	0.91 (0.77–1.09)	0.31		
**Good**	0.64 (0.53–0.76)	< 0.001	0.66 (0.50–0.86)	0.003	0.61 (0.47–0.79)	< 0.001		
**Awareness of OC symptoms**	**B) Symptoms of a nonspecific nature**							
	**Extreme generalized fatigue**		**Unexplained weight loss**		**Increased abdominal size on most days**		**Passing more urine than usual**	
	**AOR (95% CI)**	***p*-value**	**AOR (95% CI)**	***p*-value**	**AOR (95% CI)**	***p*-value**	**AOR (95% CI)**	***p*-value**
**Poor**	Ref	Ref	Ref	Ref	Ref	Ref	Ref	Ref
**Fair**	0.81 (0.72–0.92)	0.001	0.74 (0.66–0.84)	< 0.001	0.66 (0.58–0.77)	< 0.001	0.75 (0.66–0.85)	< 0.001
**Good**	0.56 (0.48–0.66)	< 0.001	0.55 (0.47–0.65)	< 0.001	0.33 (0.26–0.42)	< 0.001	0.53 (0.44–0.63)	< 0.001
**Awareness of OC symptoms**	**Changes in bowel habit**		**Persistent bloating**		**Difficulty eating on most days**		**Feeling full persistently**	
	**AOR (95% CI)**	***p*-value**	**AOR (95% CI)**	***p*-value**	**AOR (95% CI)**	***p*-value**	**AOR (95% CI)**	***p*-value**
**Poor**	Ref	Ref	Ref	Ref	Ref	Ref	Ref	Ref
**Fair**	0.87 (0.77–0.99)	0.031	0.95 (0.84–1.08)	0.44	0.80 (0.71–0.90)	< 0.001	1.01 (0.86–1.19)	0.88
**Good**	0.55 (0.47–0.65)	< 0.001	0.46 (0.39–0.55)	< 0.001	0.60 (0.51–0.70)	< 0.001	0.44 (0.37–0.53)	< 0.001

*AOR* adjusted odds ratio, *CI* confidence interval, *OC* ovarian cancer

- All analyses were adjusted for age-group, educational level, occupation, monthly income, marital status, residency, having a chronic disease, knowing someone with cancer, and site of data collection

- The outcome in all models is binary, where answers with ‘never’ were considered as ‘yes’ and all other answers were considered as ‘no’

### Association between OC symptom awareness and seeking medical advice within a week

For OC symptoms with pain, there was an associated increase in the likelihood of seeking medical advice within a week for ‘persistent low back pain’ and ‘persistent pain in the pelvis’ among participants who displayed good awareness of OC symptoms (Table 3). For non-specific OC symptoms, there was an associated increase in the likelihood of seeking medical advice within a week for five out of eight symptoms among participants with fair awareness and a further increase in the likelihood of seeking medical advice for all non-specific OC symptoms among participants with good awareness.

**Table 3:** Multivariable logistic regression analyzing the association between seeking medical advice within a week and awareness of ovarian cancer symptoms

**Awareness of OC symptoms**	**A) Symptoms with pain**							
	**Persistent low back pain**		**Persistent pain in the pelvis**		**Persistent pain in the abdomen**			
	**AOR (95% CI)**	***p*-value**	**AOR (95% CI)**	***p*-value**	**AOR (95% CI)**	***p*-value**		
**Poor**	Ref	Ref	Ref	Ref	Ref	Ref		
**Fair**	1.05 (0.93–1.19)	0.42	0.87 (0.74–1.02)	0.08	0.93 (0.80–1.09)	0.39		
**Good**	1.46 (1.23–1.74)	< 0.001	1.26 (1.00–1.59)	0.046	1.20 (0.97–1.50)	0.09		
**Awareness of OC symptoms**	**B) Symptoms of a nonspecific nature**							
	**Extreme generalized fatigue**		**Unexplained weight loss**		**Increased abdominal size on most days**		**Passing more urine than usual**	
	**AOR (95% CI)**	***p*-value**	**AOR (95% CI)**	***p*-value**	**AOR (95% CI)**	***p*-value**	**AOR (95% CI)**	***p*-value**
**Poor**	Ref	Ref	Ref	Ref	Ref	Ref	Ref	Ref
**Fair**	1.31 (1.15–1.48)	< 0.001	1.21 (1.07–1.38)	0.003	1.22 (1.07–1.39)	0.003	1.27 (1.12–1.43)	< 0.001
**Good**	1.76 (1.49–2.07)	< 0.001	1.70 (1.45–2.00)	< 0.001	2.36 (1.94–2.87)	< 0.001	1.77 (1.49–2.09)	< 0.001
**Awareness of OC symptoms**	**Changes in bowel habit**		**Persistent bloating**		**Difficulty eating on most days**		**Feeling full persistently**	
	**AOR (95% CI)**	***p*-value**	**AOR (95% CI)**	***p*-value**	**AOR (95% CI)**	***p*-value**	**AOR (95% CI)**	***p*-value**
**Poor**	Ref	Ref	Ref	Ref	Ref	Ref	Ref	Ref
**Fair**	1.12 (0.99–1.26)	0.07	1.00 (0.88–1.13)	0.96	1.14 (1.01–1.29)	0.033	0.99 (0.83–1.17)	0.89
**Good**	1.71 (1.45–2.01)	< 0.001	1.99 (1.69–2.35)	< 0.001	1.56 (1.33–1.83)	< 0.001	2.27 (1.87–2.75)	< 0.001

*AOR* adjusted odds ratio, *CI* confidence interval, *OC* ovarian cancer

- All analyses were adjusted for age-group, educational level, occupation, monthly income, marital status, residency, having a chronic disease, knowing someone with cancer, and site of data collection

- The outcome in all models is binary, where answers with ‘1–7 days’ were considered as ‘yes’ and all other answers were considered as ‘no’

### Barriers to early presentation and association with OC symptom awareness

When participants were asked if they would delay the visit to the doctor, 679 (12.5%) answered with ‘yes’ and reported at least one barrier to early presentation with emotional barriers being the most common, followed by service and practical barriers. The most frequent emotional barrier was ‘feeling worried about what a doctor might find’ (*n* = 383, 56.4%) (Table 4). ‘There is a small number of qualified female doctors in the Palestinian Ministry of Health facilities’ (*n* = 179, 26.4%) and ‘would try some herbs first’ (*n* = 375, 55.2%) were the most frequent service-related and practical barriers, respectively. All these findings were consistent in both the Gaza Strip and the WBJ. On the multivariable analysis, displaying good OC symptom awareness was associated with lower likelihood of reporting ≥ 4 emotional barriers (Table 5).

**Table 4:** Summary of the reported barriers to early presentation among study participants

Category	Barrier	Total (*n* = 679) n (%)	Gaza Strip (*n* = 282) n (%)	WBJ (*n* = 397) n (%)	*p*-value
**Emotional**	Feeling worried about what a doctor might find	383 (56.4)	159 (56.4)	224 (56.4)	0.99
	Feeling scared	368 (54.2)	156 (55.3)	212 (53.4)	0.64
	Disliking the visit to healthcare facilities	354 (52.1)	134 (47.5)	220 (55.4)	0.043
	God is the ultimate healer so there in no need to go to the doctor	310 (45.7)	128 (45.4)	182 (45.8)	0.94
	Would prefer not to know the reason of the symptom	291 (42.9)	116 (41.1)	175 (44.1)	0.48
	Would not feel comfortable to be examined by a male doctor	254 (37.4)	101 (35.8)	153 (38.5)	0.52
	Would not trust the doctor	209 (30.8)	88 (31.2)	121 (30.5)	0.87
	Embarrassment	175 (25.8)	79 (28.0)	96 (24.2)	0.29
	Would not feel confident talking about symptom with	112 (16.5)	53 (18.8)	59 (14.9)	0.17
**Service**	The small number of female doctors in the Palestinian Ministry of Health facilities	179 (26.4)	78 (27.7)	101 (25.4)	0.54
	Difficulty making an appointment with the doctor	151 (22.2)	66 (23.4)	85 (21.4)	0.57
	Feeling worried about wasting doctor’s time	136 (20.0)	51 (18.1)	85 (21.4)	0.33
	Difficulty talking to doctor	115 (16.9)	46 (16.3)	69 (17.4)	0.76
**Practical**	Would try some herbs first	375 (55.2)	132 (46.8)	243 (61.2)	< 0.001
	Has other things to do	351 (51.7)	144 (51.1)	207 (52.1)	0.82
	Would think that symptom is not something important to see the doctor for	349 (51.4)	135 (47.9)	214 (53.9)	0.14
	Too busy to go to the doctor	332 (48.9)	130 (46.1)	202 (50.9)	0.24
	Financial hardship that will not allow seeing the doctor	257 (37.9)	137 (48.6)	120 (30.2)	< 0.001
	Difficulty arranging transports to the doctor	193 (28.4)	109 (38.7)	84 (21.2)	< 0.001
	Family or husband would refuse their wife being examined by a male doctor	85 (12.5)	37 (13.1)	48 (12.1)	0.72
	Family or husband would refuse the visit to the doctor	51 (7.5)	27 (9.6)	24 (6.0)	0.10

*n* number of participants, *WBJ* West Bank and Jerusalem

**Table 5:** Multivariable logistic regression analyzing the association between having at least the median number of barriers to seek medical advice for a possible ovarian cancer symptom and participant characteristics

Characteristic	All barriers		Emotional barriers		Service barriers		Practical barriers	
	**AOR (95% CI)**	***p*-value**	**AOR (95% CI)**	***p*-value**	**AOR (95% CI)**	***p*-value**	**AOR (95% CI)**	***p*-value**
**Awareness of OC symptoms**								
Poor	Ref	Ref	Ref	Ref	Ref	Ref	Ref	Ref
Fair	1.21 (0.86–1.72)	0.28	1.03 (0.73–1.45)	0.87	0.91 (0.65–1.29)	0.61	1.43 (1.00–2.03)	0.049
Good	1.36 (0.84–2.21)	0.21	0.61 (0.38–0.98)	0.040	1.05 (0.65–1.68)	0.86	1.39 (0.85–2.26)	0.19

*COR* crude odds ratio,* AOR* adjusted odds ratio,* CI* confidence interval, *WBJ* West Bank and Jerusalem, *OC* ovarian cancer

- All analyses were adjusted for age-group, educational level, occupation, monthly income, marital status, residency, having a chronic disease, knowing someone with cancer, and site of data collection

- The outcome in all models is binary with the median number of barriers in each category utilized as the cutoff

## Discussion

In this study, the proportion of participants who would seek medical advice immediately for a possible OC symptom among Palestinian women was found to be low. Participants with better awareness of OC symptoms were more likely to seek medical advice for OC symptoms earlier. Emotional barriers were the most common barriers followed by practical and service-related barriers. Patients with good OC symptom awareness had a higher likelihood of displaying fewer emotional barriers.

The current lack of reliable screening methods for OC makes recognition of OC symptoms and early presentation key factors to improve OC mortality and morbidity [5, 31]. Therefore, facilitation of early presentation of women experiencing possible OC symptoms is crucial [6]. One important step to achieving this is to identify and address existing barriers that might impede women to seek medical help for possible OC symptoms. These barriers could be emotional, practical, service-related, or due to low perception of symptoms. This study sheds light on this with the aim of informing future educational interventions on what barriers that need to be mitigated.

### Immediate seeking of medical advice for a possible OC symptom

A previous study from the United States demonstrated that the most frequently reported OC symptoms were abdominal bloating or gastrointestinal symptoms, whereas gynecologic symptoms were least reported [32]. In this study,** ‘**increased abdominal size on most days’ was the most commonly reported symptom for immediate medical advice seeking (15.8%), which might be due to women showing good knowledge of it as a possible OC symptom [6]. In a previous study from Oman that surveyed 499 women, the shortest anticipated time of two weeks was noted for symptoms associated with blood, such as vaginal bleeding, post-coital bleeding, and postmenopausal bleeding [26]. Another study from the United Kingdom found that the shortest time for help-seeking was for persistent abdominal pain [29]. The results of this study also suggest that the nature of symptoms may play a role in the time interval from recognizing the symptoms until seeking medical advice, where varying proportions of study participants reported immediate help-seeking depending on the nature of the symptom recognized.

### Association between OC symptom awareness and seeking medical advice

Accurate recognition may be especially difficult due to the vague and non-specific nature of most OC symptoms, which may be also associated with benign conditions [5, 6]. Low awareness of cancer signs and symptoms was found to delay patient presentation [30]**,** which is consistent with this study, where participants with better awareness of OC symptoms were more likely to seek medical advice for OC symptoms earlier. The ‘*Be Clear on Cancer'* initiative was started in the United Kingdom in 2011. It is a series of awareness campaigns with the aim of promoting knowledge of cancer signs and symptoms in order to encourage earlier presentation and thus, earlier diagnosis [33]. In low- and middle-income settings, like Palestine, many pressures on health systems might lead to a lack of time and funds to spend on patient education and health promotion, which may have contributed to the existing knowledge gap. However, learning from other countries' experiences and investing in such initiatives may result in better tackling of OC as a health problem to lower the associated morbidity and mortality on the long-term.

### Barriers to early presentation and association with OC symptom awareness

A previous international study showed that the presence of different barriers was strongly associated with longer times to help-seeking [27]. Major factors related to delays in cancer presentation include ability to recognize and interpret symptoms, embarrassment and fear to have a consultation, and negative beliefs associated with a possible cancer diagnosis [34, 35]. In line with this study, Elshami and colleagues reported that the most commonly reported barriers to seeking medical advice in the Gaza Strip were emotional, and the most common barrier leading to a delay in help-seeking was ‘feeling worried about what the doctor might find’ [34]. Fear is an important barrier especially in low- and middle-income countries, which may be attributed to experiences of high mortality rates [36]. On the other hand, practical barriers were reported to be more important in high-income countries [37, 38]. Moreover, negative beliefs, such as cancer treatment being worse than the cancer itself and cancer diagnosis as a death sentence, may play a role in delaying patient presentation [28]. Furthermore, shortages of medicines and lack of access to cancer treatments in Palestine due to movement restrictions, especially when exiting the Gaza Strip, may contribute further to the worsening access to effective cancer treatment and, thus, having a negative impact on the outcomes [34]. Therefore, it is especially critical to develop strategies to avoid delays in diagnosis as well as to improve access to cancer treatment in low- resource settings. Education campaigns have been reported to increase cancer knowledge and awareness, such as a video educational program from Arizona that led to increased awareness of cancer symptoms [39] or the Inside Knowledge campaign that increased OC awareness as well as providers' confidence in discussing OC symptoms [31]. Ultimately, those interventions led to earlier detection and improved management of OC.

### Future directions

Awareness campaigns about OC symptoms and barriers to early presentation could lead to earlier seeking of medical advice and, thus, better overall outcomes in OC treatment. A multidisciplinary and culturally adapted approach should be used to enlighten women about OC and help them rationalize the fears and false presumptions regarding the disease. The use of multiple platforms for delivery of such content could be beneficial to widen its reach, including traditional print media, posters, screens in waiting rooms of healthcare facilities, TV and radio as well as social media [33, 39]. Social media could be especially helpful as it facilitates communications between patients, providers, and health policy makers. It enables better access to health information making it an excellent tool in disseminating cancer prevention, screening, and treatment messages to the community. Social media also provides patients and the general population with the opportunity to interact with clinicians beyond their geographic location and allow them to build virtual communities that facilitate online discussions regularly.

Training more female physicians and improving their communication skills are needed to help addressing the concerns that Palestinian women may have about the availability of enough trained female physicians. This is especially important as discussions about OC might be perceived culturally as a sensitive subject.

### Limitations

Some limitations should be considered when interpreting the findings of this study. The convenience sampling method employed may limit the generalizability of the study findings to the entire population of Palestinian women. However, the large sample size, high response rate, and recruitment from various geographic locations might have mitigated this. Furthermore, the low number of women aged 65 and over reflects that a small proportion of women at the highest risk of OC was included in the study. However, this relatively small proportion reflects the small section of the female population who is 65 years old or over in Palestine [40]. Moreover, this study examined women’s perceptions if they ‘might experience’ such symptoms and possible behavior as opposed to women who actually experience OC symptoms and their actual behavior, which could be different.

## Conclusion

A low proportion of Palestinian women would seek medical advice immediately if they recognized a possible OC symptom. Women with higher awareness of OC symptoms were more likely to seek help sooner. The most common barriers to seeking help were emotional followed by practical and service-related barriers. Women who were more aware of OC symptoms had a lower likelihood of reporting emotional barriers. The findings of this study suggest that health policymakers should focus on increasing awareness of OC symptoms and reducing emotional barriers to seeking help in order to improve early diagnosis and treatment. This could be done through public education campaigns, training of more female healthcare providers, and establishing support groups for women with OC.

### Supplementary Information

**Additional file 1:** **Supplementary table 1.** Characteristics of study participants.

## Data Availability

The dataset used and analyzed during the current study is available from the corresponding author on reasonable request.

## Abbreviations

OC: Ovarian cancer
WBJ: West Bank and Jerusalem
OCAM: Ovarian cancer awareness measure
CI: Confidence interval
OR: Odds ratio
