# Supplementary material for: Anticipated time to seek medical advice for possible ovarian cancer symptoms and perceived barriers to early presentation among Palestinian women: a national cross-sectional study
Source: BMC Cancer. 2023 Oct 13;23:975. doi: 10.1186/s12885-023-11484-z (PMC10571332; doi:10.1186/s12885-023-11484-z)
Supplement: Supplementary file 1 — Additional file 1: Supplementary table 1. Characteristics of study participants. [file 12885_2023_11484_MOESM1_ESM.docx]

**Anticipated Time to Seek Medical Advice for Possible Ovarian Cancer Symptoms and Perceived Barriers to Early Presentation among Palestinian Women: A National Cross-sectional Study**

**Authors:**

Mohamedraed Elshami, MD, MMSc^1,2^*, Sondos Al-Madhoun, MD^3^*, Mohammed Alser, MD^4^, Ibrahim Al-Slaibi, MD^5^ Areej Yaseen^6^, Aya Tuffaha, MD^7^, Hadeel Jabr, MD^2^, Sara Ubaiat^8^, Salma Khader^4^, Reem Khraishi^9^, Inas Jaber, MD^6^, Zeina Abu Arafeh^6^, Aya Alqattaa^10^, Asmaa Abd El Hadi^10^, Ola Barhoush^4^, Maysun Hijazy^10^, Tamara Eleyan^4^, Amany Alser^10^, Amal Abu Hziema^10^, Amany Shatat^10^, Falasteen Almakhtoob^11^, Balqees Mohamad, MD^12^, Walaa Farhat^13^, Yasmeen Abuamra^14^, Hanaa Mousa^10^, Reem Adawi^6^, Alaa Musallam, MD^15^, Shurouq I. Albarqi, PharmB^16^, Nasser Abu-El-Noor, PhD^17#^, Bettina Bottcher, MD, PhD^10#^

*Contributed equally as a first co-author.

^#^Contributed equally as a senior co-author.

^1^Division of Surgical Oncology, Department of Surgery, University Hospitals Cleveland Medical Center, Cleveland, OH, USA.
^2^Ministry of Health, Gaza, Palestine.
^3^Al-Shifa Hospital, Gaza, Palestine.

^4^United Nations Relief and Works Agency for Palestine Refugees (UNRWA), Gaza, Palestine.
^5^Almakassed Hospital, Jerusalem, Palestine.

^6^Faculty of Medicine, Al-Quds University, Jerusalem, Palestine.

^7^Al-Watani Hospital, Nablus, Palestine. ^8^Faculty of Medicine, Al-Quds University, Bethlehem, Palestine.

^9^Faculty of Medicine, An-Najah National University, Nablus, Palestine.

^10^Faculty of Medicine, Islamic University of Gaza, Gaza, Palestine.
^11^Faculty of Medicine, Palestine Polytechnic University, Hebron, Palestine.
^12^Doctors Without Borders (Medecins Sans Frontieres), Hebron, Palestine.
^13^Faculty of Medicine, Al-Quds University, Jenin, Palestine. ^14^Faculty of Medicine, Al-Azhar University of Gaza, Gaza, Palestine.

^15^Al-Aqsa Hospital, Deir Albalah, Palestine.

^16^Faculty of Pharmacy, Al-Azhar University of Gaza, Gaza, Palestine. ^17^Faculty of Nursing, Islamic University of Gaza, Gaza, Palestine.

**Corresponding author**

Mohamedraed Elshami, MD, MMSc

Division of Surgical Oncology

Department of Surgery

University Hospitals Cleveland Medical Center

11100 Euclid Avenue, Lakeside 7100

Cleveland, OH 44106
Phone: 832-245-6055

Email: mohamedraed.elshami@gmail.com

| **Supplementary table 1:** Characteristics of study participants. | | | | |
| --- | --- | --- | --- | --- |
| **Characteristic** | **Total**  **(n= 5411)** | **Gaza Strip**  **(n= 2278)** | **WBJ**  **(n= 3133)** | **p-value** |
| **Awareness of OC symptoms**, n (%)  Poor  Fair  Good | 1943 (35.9)  2525 (46.7)  943 (17.4) | 780 (34.2)  1020 (44.8)  478 (21.0) | 1163 (37.1)  1505 (48.0)  465 (14.8) | <0.001 |
| **Mean age± SD** | 32.0 **±** 12.3 | 33.2**±** 11.2 | 36.1**±** 13.0 | <0.001 |
| **Age group,** n (%)  18 to 44  45 or older | 4151 (76.7)  1260 (23.3) | 1872 (82.2)  406 (17.8) | 2279 (72.7)  854 (27.3) | <0.001 |
| **Menarche,** n (%)  Early (≤ 10 years)  Normal (11-15 years)  Late (≥ 16 years) | 65 (1.2)  4658 (86.1)  688 (12.7) | 20 (0.9)  1923 (84.4)  335 (14.7) | 45 (1.4)  2735 (87.3)  353 (11.3) | <0.001 |
| **Educational level,** n (%)  Secondary or below  Post-secondary | 3016 (55.7)  2395 (44.3) | 1330 (58.4)  948 (41.6) | 1686 (53.8)  1447 (46.2) | <0.001 |
| **Occupation,** n (%)  Unemployed/housewife  Employed  Retired  Student | 3671 (67.8)  1095 (20.2)  47 (0.9)  598 (11.1) | 1837 (80.6)  254 (11.2)  9 (0.4)  178 (7.8) | 1834 (58.5)  841 (26.8)  38 (1.2)  420 (13.4) | <0.001 |
| **Monthly income ≥ 1450 NIS,** n (%) | 3330 (61.5) | 474 (20.8) | 2856 (91.2) | <0.001 |
| **Marital status,** n (%)  Single  Married  Divorced/Widowed | 1248 (23.1)  3952 (73.0)  211 (3.9) | 374 (16.4)  1836 (80.6)  68 (3.0) | 874 (27.9)  2116 (67.5)  143 (4.6) | <0.001 |
| **Having a chronic disease,** n (%) | 1097 (20.3) | 350 (15.4) | 747 (23.8) | <0.001 |
| **Knowing someone with cancer,** n (%) | 2746 (50.8) | 1104 (48.5) | 1642 (52.4) | 0.004 |
| **Site of data collection,** n (%)  Public Spaces  Hospitals  Primary healthcare centers | 1645 (30.4)  1735 (32.1)  2031 (37.5) | 596 (26.2)  650 (28.5)  1032 (45.3) | 1049 (33.5)  1085 (34.6)  999 (31.9) | <0.001 |
| n= number of participants, SD= standard deviation, WBJ= West Bank and Jerusalem, OC= ovarian cancer. | | | | |
